# Residual Endodontic Filling Material after Post Space Preparation: A Confocal Microscopic Study

**DOI:** 10.3390/ma10111333

**Published:** 2017-11-21

**Authors:** Yu-Yao Teoh, Laurence J. Walsh

**Affiliations:** School of Dentistry, The University of Queensland, Herston, Brisbane QLD 4006, Australia; l.walsh@uq.edu.au

**Keywords:** sealer, cement, post space preparation, confocal microscopy, MTA, calcium hydroxide

## Abstract

This laboratory study assessed removability of endodontic alkaline cements and resin sealers using coronal cross-sectional slices of roots with single canals. Materials were labelled with 0.1% (*w*/*w*) sodium fluorescein prior to mixing so that confocal microscopy could be used to quantify material remaining on the walls of post spaces, to assess cleanliness. Roots of extracted teeth were prepared using rotary NiTi instruments then obturated using lateral condensation with gutta percha and epoxy resin sealers (AH-Plus™ or Zirmix™), or were filled by injecting mineral trioxide aggregate (MTA) cement (GC Nex™ MTA or MTAmix™) or a hard-setting calcium hydroxide cement (Supercal™). Brown (#3) ParaPost™ drills were used at 600 rpm with a torque setting of 3 N cm^−1^ for 2 min to remove 5 mm of the root filling. Roots were embedded and coronal slices examined by confocal microscopy, with the perimeter of the drill channel divided into clean, unclean and non-accessible regions. The choice of material affects cleanliness, with MTA being the most difficult and calcium hydroxide cement the easiest to remove. With epoxy resin-based sealers, almost half of the accessible canal walls remained coated with remnants of sealer after post space preparation.

## 1. Introduction

Teeth requiring root canal treatment have often lost considerable coronal tooth structure, and therefore are restored with a crown, which may be supported by a post and core, with the post placed into the root canal space. A typical post space is a parallel sided preparation with a wall thickness of up to 1 mm of radicular dentine [1]. Beneath the post space, some 3–5 mm of the root canal filling material is left in place at the apical third region [2]. The depth of the post space varies according to the required length of the post and the root morphology. Post retention is greater with longer posts [3]. Posts may be rigid or flexible. Rigid posts are constructed from a metal alloy, while flexible posts can be made from various types of ceramics or fiber materials.

To prepare the post space, removal of root canal filling material is most often done using rotary instruments such as Gates-Glidden burs and parallel sided post drills. Such instruments may be used in conjunction with solvents such as chloroform to soften the gutta-percha (GP). Posts are typically retained in the root by cementation or bonding to the dentine walls of the post space [4]. For effective cementation, the walls of the post space prepared in the canal must be clean [5]. The post space preparation should have walls that are dentine, to facilitate post cementation with adhesive materials such as resin cements which rely on the formation of a hybrid zone between the resin cement and dentinal tubules [6,7]. There should be no remnants of root canal filling materials, since these cannot give reliable cementation or bonding [8,9]. Canal enlargement beyond the initial preparation size reduces residues of the filling material on the canal walls [10]. To minimise radicular dentine removal and help preserve the fracture strength of the tooth, the post drill should match the prepared canal size.

Previous studies have assessed removal of root filling materials using micro-computed-tomography (micro-CT) [11,12,13]. Roggendorf et al. measured the percentage of sealer residue on the canal surface using micro-computer-tomography but this methodology was extremely time consuming and expensive [13]. Other studies involving micro-CT have employed volumetric analysis [11,12]; however, a large reduction in the volume of the root filling material does not necessarily correlate to the cleanliness of the canal walls. Studies utilising scanning electron microscopy [14] or stereomicroscopy [15] only examine a small portion of the circumference of the walls of the post space.

There has been recent interest in the use of alkaline cements as a root filling material for their antimicrobial properties and dimensional stability [16]. The manufacturers of these materials advocate a bulk filling technique without the need of GP cones. Consequently, the way in which these materials are removed to create a post space may differ from conventional techniques involving GP and epoxy resin sealer.

The present study examined cross-sectional slices of roots and used confocal microscopy to quantify remnants of material on the perimeter of the post space, using fluorescence labelling. The study compared the cleanliness of canal walls after post space preparation of conventional GP-epoxy resin root fillings with alkaline cements.

## 2. Materials and Methods

### 2.1. Collection and Preparation of Teeth

A total of 80 extracted human permanent teeth with a single root canal were collected from an oral surgery clinic with the approval of the institutional ethics committee (Approval code #1311). After removing the crowns at the cemento-enamel junction, the root face was polished to form a flat coronal surface using abrasive discs. After patency of the root canals was confirmed using a #8 K-file (Dentsply Maillefer, Ballaigues, Switzerland), the canals were prepared with nickel-titanium rotary instruments (ProTaper Next™, Dentsply Maillefer, Ballaigues, Switzerland) to size X3 with variable taper and an apical preparation size of ISO #30. During instrumentation the canals were irrigated alternately with 1% *w*/*v* sodium hypochlorite (Endosure Hypochlor 1% Solution™, Dentalife, Ringwood, Melbourne, Australia) and 15% *w*/*v* ethylenediaminetetraacetic acid (EDTA) with 0.85 *w*/*v* cetrimide (Endosure EDTA/C 15% Solution™, Dentalife, Ringwood, Melbourne, Australia) using syringes with side-vented needles. After a final irrigation step using EDTA for 2 min [17], the canals were dried with paper points, and the roots stored in saline at room temperature.

### 2.2. Experimental Groups

The roots were assigned randomly into 5 groups of 16 each. The root canal sealers and cements were tagged with sodium fluorescein to a final concentration of 0.1% (*w*/*w*) prior to mixing. This concentration was chosen to be as low as possible to give sufficient labelling without diluting the components of the sealers or cements. Sodium fluorescein is stable at a broad range of pH values, and has low chemical reactivity. According to the protocols detailed in Table 1, below, one of five different materials were used to fill the prepared root canals. AH-Plus™ and Zirmix™ epoxy resin sealers were used to obturate the canal in combination with gutta percha (GP) points using the lateral condensation technique. A ProTaper Next™ X3 GP point (Dentsply Maillefer, Ballaigues, Switzerland) with an ISO #30 apical size and variable taper was used as a master cone followed by medium and fine accessory points placed with the aid of a finger spreader.

Nex MTA™ (grey MTA), MTAmix™ (white MTA) and Supercal™ were used as bulk obturating materials. The materials were hand-mixed according to manufacturer’s instructions, and then injected into the canal using a syringe until excess material was seen to extrude apically.

### 2.3. Storage of Samples

Complete obturation of all roots was confirmed radiographically using X-ray exposure settings of 65 kV at 7 mA for 0.16 s. The filled roots were stored in a 100% humid environment at 37 °C for 90 days to ensure complete setting of all materials had occurred under simulated oral conditions. This approach had previously been employed in a study of bacterial penetration through the same filled roots over 90 days [16].

### 2.4. Removal Procedures

**Figure 1:**
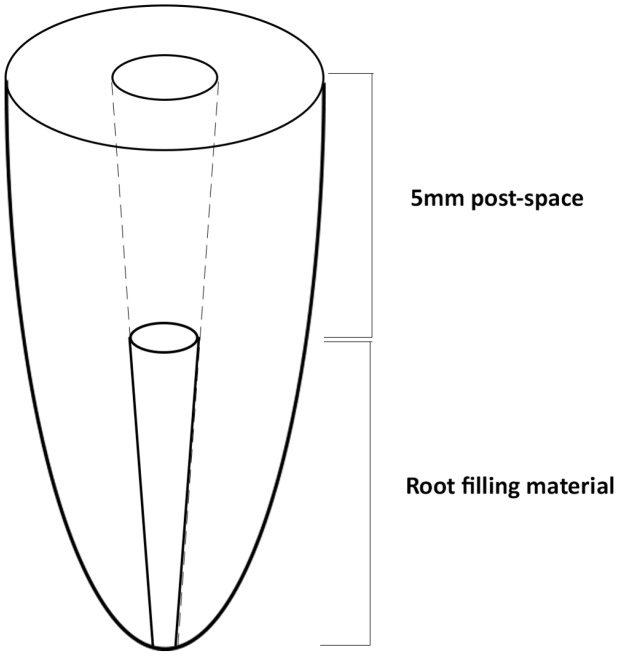
Schematic diagram of post space preparation.

Brown (#3/0.9 mm) ParaPost™ drills (Coltene-Whaledent Inc., Cuyahoga Falls, OH, USA) were attached to an endodontic motor (X-Smart™, Dentsply Maillefer, Ballaigues, Switzerland) and operated at 600 rpm with a torque setting of 3 N·cm^−1^ for a fixed time of 2 min to remove 5 mm of material, measured from the coronal surface (Figure 1). A drill smaller than the prepared canal size was chosen to minimise dentine removal, as the aim of the study was to assess the removability of root canal filling material from canal wall. In eccentric canals, the drill was tilted laterally until contact with the canal wall on the coronal surface was achieved. The handpiece was then moved in a clockwise direction to allow the sides of the the drill to contact the canal walls. Roots were prepared in a random sequence by a single operator who used ×2.5 magnification loupes. Drills were changed after every 5 samples. The prepared post space was flushed with saline and dried with paper points.

**Figure 2:**
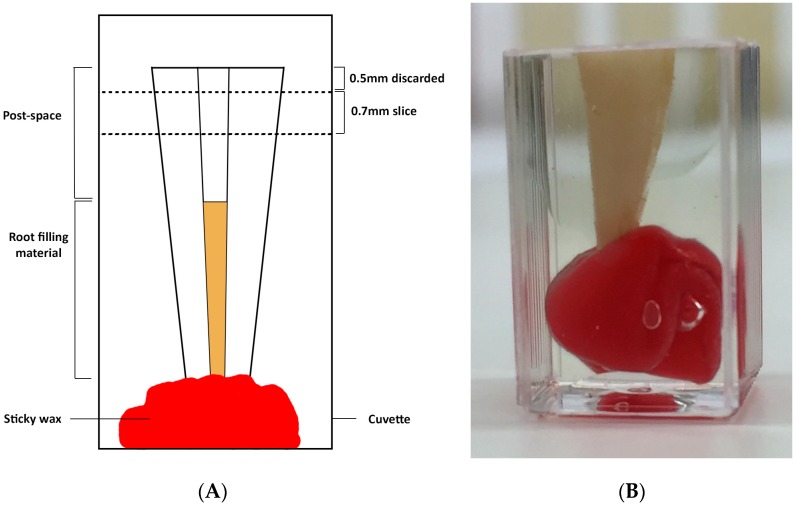
(**A**) Schematic diagram of a root mounted in a cuvette with the location of the 0.7 mm slice shown; (**B**) The sample after removing 0.5 mm from the coronal surface, just before taking the 0.7 mm slice.

The roots were then placed upright into a standard clear plastic spectrophotometer cuvette and held in place with wax attached at the apex. Clear epoxy resin (Presi MA2+™, Kemet, Marayong, NSW, Australia) was introduced into the root canal with an irrigating needle and syringe to prevent voids in the post space, and the cuvette then filled with the same epoxy resin to submerge the root. After allowing the resin to set for 24 hours at room temperature according to the manufacturer’s instructions, the embedded roots were sectioned horizontally using a diamond saw (IsoMet™ 1000, Buehler, Lake Bluff, IL, USA). A 0.7 mm thick slice was taken 0.5 mm below the coronal surface (Figure 2).

**Figure 3:**
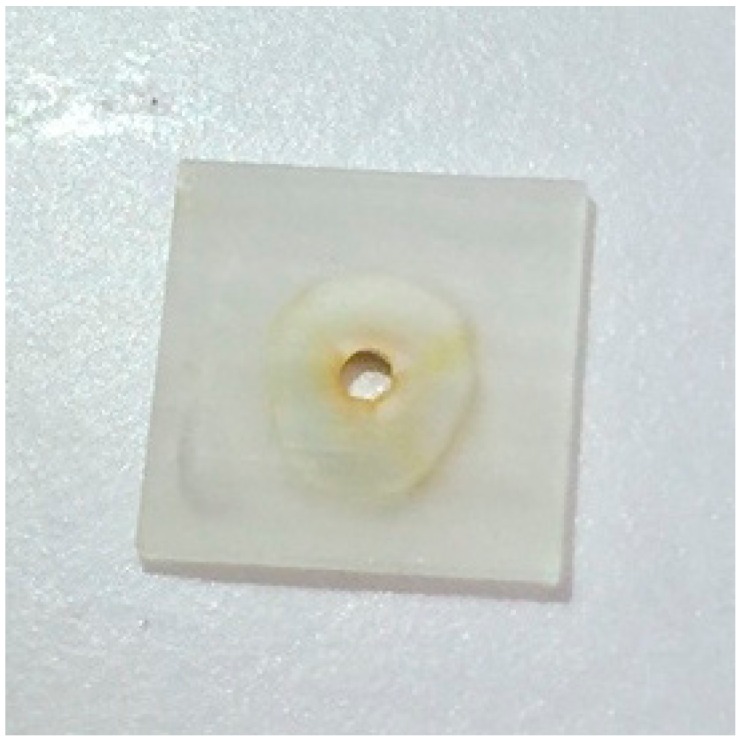
Typical 0.7 mm thick cross-sectional slice of a root with the post space prepared.

The coronal root slices (Figure 3) were examined under a confocal laser scanning microscope (CLSM) (Model C2+, Nikon Instruments Inc., Melville, NY, USA) using 488 nm wavelength laser excitation. Samples were imaged using a ×10 objective lens, and high-resolution composite images made to encompass the entire cross-sectional area of the root.

**Figure 4:**
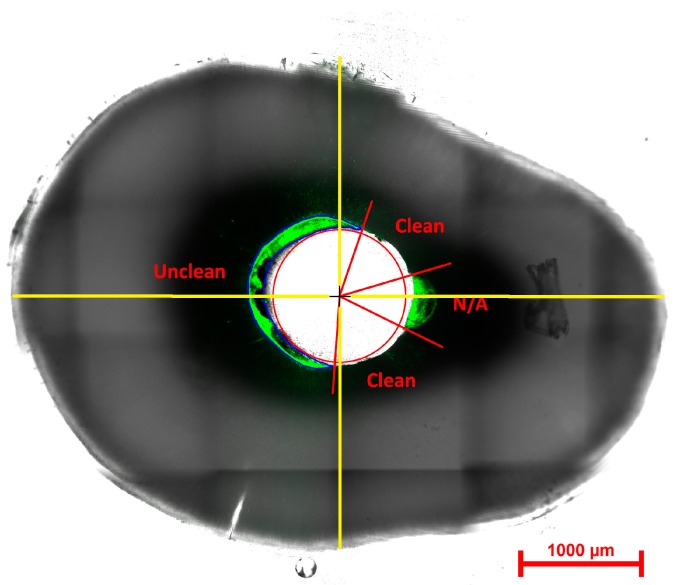
Composite bright field and fluorescence image showing the method for assessing canal walls for remnant sealer or cement and measuring eccentricity. The red circle shows the outline of the ParaPost™ drill. The centre of this circle marked with a crosshair was used to align the protractor for angular measurements. Remaining GP appears as a shadow outlined in blue. Regions on the circumference of the drill were classified as clean, unclean or non-accessible. Green areas are sodium fluorescein-tagged remnants of sealer or cement on the canal walls, marked as unclean. Areas designated as non-accessible (N/A) cannot be reached by the ParaPost™ drill. The yellow lines crossing perpendicularly at the crosshair are the major axis and minor axis lengths taken to calculate eccentricity.

Eccentric regions, isthmuses and lateral fins that could not be accessed by the drill were identified, and the angular distribution of clean walls and walls with material remaining along the perimeter of the drill channel measured in degrees using a protractor placed at the centre of the drill position (Figure 4). For this purpose, a protractor was printed on transparency film and overlaid onto the display monitor. The cleanliness of the wall was calculated as a percentage of the angle, which could be instrumented or accessed by the ParaPost™ drill, thereby excluding the non-accessible areas.

### 2.5. Statistical Analysis

Statistical analysis was performed using Prism™ software version 7.0a (GraphPad Software Inc., La Jolla, CA, USA). As the data sets for MTAmix™ and Supercal™ did not show a Gaussian distribution using the D’Agostino & Pearson normality test, differences between groups were compared using the non-parametric Kruskal-Wallis test followed by post-hoc Dunn’s tests. The threshold for statistical significance was set at *p* < 0.05.

### 2.6. Eccentricity

Eccentricity is the measure of how much a cross section (e.g., an ellipse) varies from being circular, with the eccentricity of a circle being zero [18]. Based on the centre point of the root canal, the major (a) and minor (b) axis lengths of the root were determined, as shown in Figure 4. The lengths of the major and minor axes were measured (in μm) using the Nikon confocal microscope software. The eccentricity calculation was based on the ratios of the length of the major axis to the minor axis, according to the following equation.

$$\mathrm{Eccentricity}\;=\;\sqrt{1-\frac{b^{2}}{a^{2}}};\;\mathrm{where}\;a\;=\;\mathrm{major}\;\mathrm{axis},\;b\;=\;\mathrm{minor}\;\mathrm{axis}$$

The eccentricity of each root was compared against the percentage of the angular distribution which could be instrumented or accessible areas to determine if a correlation existed between the outline of a tooth and the ability to remove root canal filling material.

## 3. Results

**Figure 5:**
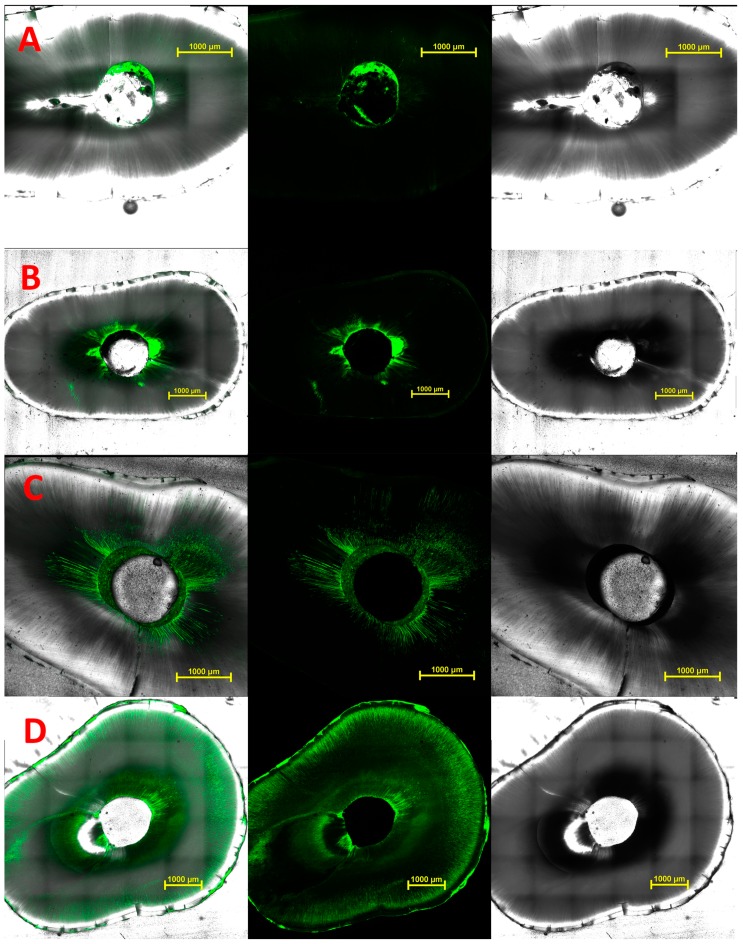
Horizontal cross-sections of roots. From left to right, superimposed bright-field image and fluorescence staining, fluorescence staining only and bright-field image only. Remnants of fluorescein-tagged sealer or cement are in green. Image sets are (**A**) AH-Plus™; (**B**) Zirmix™; (**C**) MTAmix™; and (**D**) Supercal™. Scale bars are 1 mm.

The presence of residual materials on canal walls was identified readily, since remnants showed strong green fluorescence because of the fluorescein label (Figure 5). As shown in Figure 5 panels A and B, remnants of GP could be identified as a shadow on the bright-field images. As shown in Figure 5 panel C, a thin layer of MTAmix™ could be seen coating the walls despite the large volume of the cement which had been removed from the canal.

With all products, material that was located within lateral canals or small fins was not removed by the ParaPost™ drill, hence this region was regarded as non-accessible. Removal of 5 mm of Nex MTA™ from the canal was not possible due to the inherent resistance of this material to drilling, and so this group (Group 3) was excluded from further analysis.

Data for canal wall cleanliness are shown in Table 2 below. In order of canal wall cleanliness from highest to lowest (expressed in percent), the groups were ranked as follows: Group 5 Supercal™, Group 2 Zirmix™, Group 4 MTAmix™, and Group 1 AH-Plus™.

There was a significant difference between the four groups (*p* < 0.0001) as measured using the Kruskal-Wallis test. Comparisons between groups showed that the median cleanliness of canal wall for Group 5 Supercal™ CHC was significantly higher than that for Groups 1 (*p* < 0.0001), 2 (*p* = 0.0061) and 4 (*p* = 0.006), but there were no significant differences between the epoxy resins and MTAmix™ (*p* > 0.9999).

**Figure 6:**
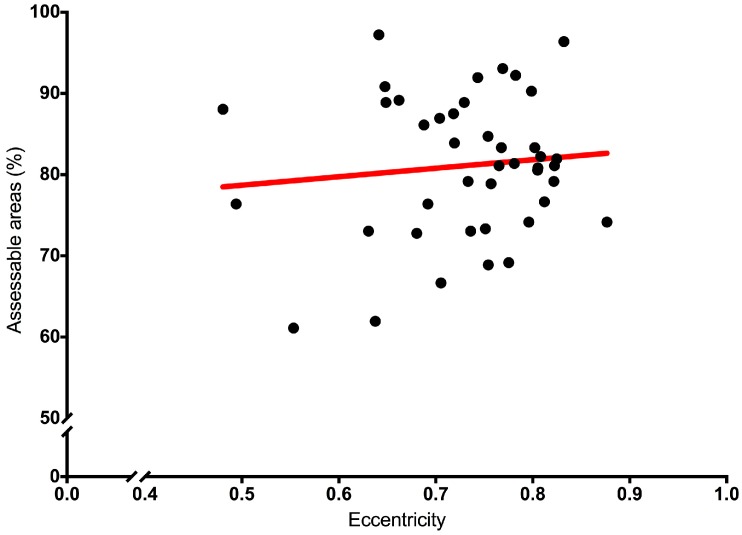
Linear regression plot of the measured eccentricity of the root versus the assessable area of a root canal of the same tooth in percentage, showing the line of best fit.

When the eccentricity of the roots was plotted against the percentage of the accessible areas of the root canal, no direct linear relationship was seen (Figure 6). These data were also assessed for non-linear correlation using the Spearman rank order test, and this also failed to show any significant relationship (*p* < 0.7493, R = 0.05082).

## 4. Discussion

The present study used confocal microscopic analysis of fluorescein-tagged materials to show remnant material on canal walls after post space preparation. The technique of labelling materials detected by confocal microscopy as used in this study appears to be a suitable and economical means to assess the cleanliness of canal walls. This approach has several advantages over scanning electron microscopy (SEM), including reliable identification of labeled materials, and simpler sample preparation. A small ParaPost™ drill was used to minimise the drill from cutting into radicular dentine, in order to focus on the ability of the drill to remove material from canal walls. Clinically, drills are chosen to match the canal in size so as to preserve radicular dentine and lower the chance of root fracture.

From the perspective of the cross-sectional circumference of the post space preparation, the hard-setting calcium hydroxide cement (Supercal™) was found to be relatively easy to remove, with less than 8% of the material remaining on accessible canal walls. Despite being a dimensionally stable polymer [16], once fully set, Supercal™ can still be removed using light force applied to a ParaPost™ drill. Further research on how this material performs as a possible alternative to the GP/epoxy resin-sealer obturation approach currently in mainstream use is needed.

MTA cements, which are based on Portland cement, cannot easily be removed using a ParaPost™ drill. Of the two MTA cements in the study, MTAmix™ was more easily removed than nexMTA™, which is a harder and more rigid material when set. MTA may be encountered during post space preparation when a tooth has had previously undergone a perforation repair, but it is unlikely to be used as a bulk root filling material. Past studies have shown that fully set MTA is difficult to remove [19]. Trying to remove this material using diamond or tungsten carbide burs carries a high risk of the drill wandering into adjacent (softer) dentine. There are no specially made solvents for MTA. Exposure to strong acids will cause MTA to degrade [20], but will also demineralise adjacent tooth structures.

In the present study, the gutta-percha epoxy resin sealer combination showed relatively poor removability with a regular ParaPost™ drill, with around half the perimeter of the accessible canal wall still coated in remnants of the sealer. Attempts to cement a post into the post space could be compromised by the residues of sealer that are still present on the walls. Some protocols for removal of GP-based root fillings employ solvents such as chloroform or eucalyptus oil, which will soften GP and epoxy resin sealers [21]. While such solvents may be used in endodontic re-treatment cases, they are usually avoided for post space preparation due to concerns that the remaining GP in the apical third of the root may shrink because of evaporation of solvent, which would compromise the seal of the remaining root filling [2,22]. Insoluble materials have been used in the past as a coronal plug to protect the apical root filling from bacterial contamination after post space preparation [23,24]. The use of a coronal plug followed by solvents in the post space may improve cleanliness and should be explored further. As the present study was focused on the removability of different materials with a ParaPost™ drill, further work is needed to determine how outcomes could differ if particular solvents were used in conjunction with the drill.

Despite the efforts to remove root canal filling materials, it is inevitable that some material remains in the canal. While rotary drills produce a circular cutting outline, the cross-sectional shape of the root canal is rarely a uniform circular shape [25]. This means that material in lateral fins and isthmuses will be inaccessible and will not be removed. The roots used in the present study did not show a large variation in their cross-sectional shape. There was a small but not statistically significant association between eccentricity and accessible area. One reason for this is that the outline of the root canal does not necessarily follow the external shape of the root, because of the presence of features such as lateral canals and isthmuses, as seen in Figure 5A. This is where new conforming endodontic files such as TRUShape™ (Dentsply Tulsa Dental Specialties, Tulsa, OK, USA), XP Endo™ finisher (FKG) and SAF™ (ReDent Nova, Ra’anana, Israel) may have advantages for the removal of obturation material. Such instruments can give improved contact with canal walls, and should reduce the extent of non-accessible areas in eccentric canals.

The present study focused on the cleanliness of canal walls after post space preparation when using a circular drill to match a preformed post, as would be done when a post space is prepared shortly after root canal treatment is completed. Effective bonding of a circular post to a root with a circular post space will be maximised when there are no remnant materials on the perimeter of the post space. The present study showed that penetration of sealers into the dentine tubules varied between materials, and this could affect later bonding as the tubules will no longer be patent. Furthermore, the use of antimicrobial agents [26], antibacterial components [27] or other irrigants [28] could alter the bonding surface of the post space. Results of the present study should be confirmed with future studies accounting for these variables.

With regard to other directions for further work, the possible influence of residues of root filling material that sit outside the circular outline of the post drill and within lateral fins and isthmuses remains to be determined. Care must be taken to preserve asepsis of the root canal space, by preventing saliva contamination during post space preparation. In contrast, for re-treatments, asepsis is not assumed and all root filling material is removed, including that in lateral fins and isthmuses.

## 5. Conclusions

The choice of material affects removability from the canal walls, with MTA being most difficult to remove, and the calcium hydroxide cement Supercal™ the easiest to remove with a ParaPost™ drill. When epoxy resin-based sealers are present, almost half of the accessible canal walls remains coated with remnants of sealer after post space preparation.

## Figures and Tables

**Table 1:** Treatment groups.

1. AH-Plus™ (Dentsply Maillefer, Ballaigues, Switzerland) non-staining epoxy resin paste/paste sealer for permanent filling of root canals with gutta-percha.
2. Zirmix™ (Ozdent, Castle Hill, Sydney, Australia) non-staining epoxy resin powder/paste root canal sealer comprised of the resin liquid component from AH26™ (Dentsply, Ballaigues, Switzerland) mixed with a powder of zirconium dioxide (80%) and hexamethylenetetramine (20%) (Wright Corporation, Wilmington, NC, USA). Bismuth trioxide in the original AH26™ powder was substituted with zirconium dioxide to prevent staining.
3. Nex MTA™ (GC Corporation, Tokyo, Japan) grey MTA cement packaged in uniform sachets and according to manufacturer, prepared by mixing with water.
4. MTAmix™ (Ozdent, Castle Hill, Sydney, Australia) white MTA cement dissolved in a solution containing glycerol and water to increase dissolution of calcium compounds and improve handling properties.
5. Supercal™ (Ozdent, Castle Hill, Sydney, Australia) hard-setting calcium hydroxide cement (CHC) that contains glycerol, calcium sulfate hemihydrate and zirconium dioxide.

**Table 2:** Descriptive statistics of percentage of canal wall cleanliness.

Group	Material	Mean	Standard Deviation	Minimum	Median	Maximum	95% Confidence Internals
1	AH Plus™	48.2	18	20.9	43.1 (A)	78.3	33.9–69.5
2	Zirmix™	64.1	18.1	43.6	61.0 (B)	100	49.1–77.9
4	MTAmix™	58.8	31.9	14	49.1 (C)	100	29.2–98.3
5	Supercal™	95.2	10.2	64.2	100	100	92.8–100.0

Statistically significant differences between groups are indicated as letters. (A) AH-Plus vs. Supercal *p* < 0.0001; (B) Zirmix vs. Supercal *p* = 0.0061; (C) MTAmix vs. Supercal *p* = 0.0006.
